# *Brevipalpus* mites (Acari: Tenuipalpidae): vectors of invasive, non-systemic cytoplasmic and nuclear viruses in plants

**DOI:** 10.1007/s10493-012-9632-z

**Published:** 2012-12-01

**Authors:** Jose Carlos Verle Rodrigues, Carl C. Childers

**Affiliations:** 1 Crops and AgroEnvironmental Sciences Department, Agricultural Experimental Station-Río Piedras, University of Puerto Rico, 1193 Calle Guayacan, San Juan, PR 00926 USA; 2 Center for Excellence in Quarantine and Invasive Species, University of Puerto Rico, Biology Bldg, 1193 Calle Guayacan, San Juan, PR 00926 USA; 3 Department of Entomology and Nematology, Citrus Research and Education Center, University of Florida, Lake Alfred, FL USA; 4 Center for Applied Tropical Ecology and Conservation (CATEC, CREST-NSF), Rio Piedras Campus Facundo Bueso Building Office 301—A, San Juan, PR 00931 USA

**Keywords:** Tenuipalpidae, Vector–virus relationships, Invasive species, *Brevipalpus phoenicis* complex

## Abstract

Multi-directional interactions occur among plant hosts, *Brevipalpus* mites and the plant viruses they transmit. Such interactions should be considered when evaluating the severity of a disease such as citrus leprosis. The current understanding of *Brevipalpus*-transmitted viruses relies on the capability of the vector to transmit the disease, the persistence of the virus in the host plant and the ability of the disease to spread. Previously, we discussed the *Citrus leprosis virus* (CiLV) and its importance and spread over the past decade into new areas of South and Central America, most recently into southern Mexico and Belize. Here, we address key questions to better understand the biology of the mite vector, fitness costs, and the peculiarities of *Brevipalpus* mite reproduction, virus survival, transmissibility and spread, and the expansion of the host plant range of *Brevipalpus* species vectoring the disease.

## Biological peculiarities of *Brevipalpus* mite vectors

Previous papers have reviewed the basic biology of several *Brevipalpus* species on citrus, tea and other plants (Oomen 1982; Haramoto 1969, Chiavegato 1986, Childers et al. 2003a, b). However, new information has come to light that species that have been previously identified as *Brevipalpus phoenicis* (Geijkes) are actually a complex of morphologically similar species (Beard et al. 2012) and biotypes which co-infest various plant species (Rodrigues et al. 2004, 2008, Kitajima et al. 2010). This requires a closer look at their biology to determine the relationship of the species within the complex that vector diseases with the invasive viruses, such as citrus leprosis and related plant viruses.

**Fig. 1:**
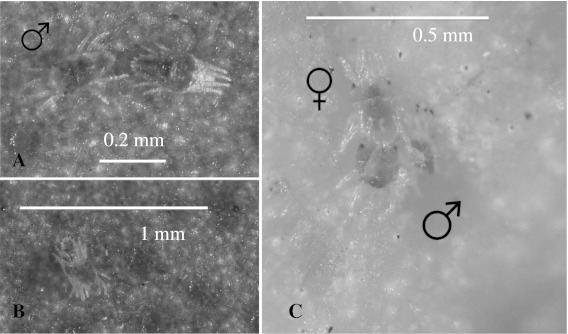
Occurrence of males (**a** left, **b** on top) *Brevipalpus phoenicis* guarding an immature female teleiochrysalis and **c** mating occurred following the emergence of the adult female

Weeks et al. (2001) found that one of the studied *B. phoenicis* populations was predominantly thelytokous, in association with the occurrence of feminizing bacteria in the genus *Cardinium*. However, in mite populations on citrus from the Tocantins State in Brazil (Domingues and Rodrigues 1999) and Florida, males have been observed guarding the immature female teleiochrysalis stage followed by mating, once the adult female emerges (Fig. 1). This same behavior had been reported in the Tetranychoidea superfamily (Collins et al. 1993). Weeks et al. (2001) found that females of *B. phoenicis* treated with antibiotics that eliminated feminizing bacteria produced higher numbers of males. It does not seem conceivable that males would expend this much output of energy in elaborate female guarding strategies if they were not sexually functional.

**Fig. 2:**
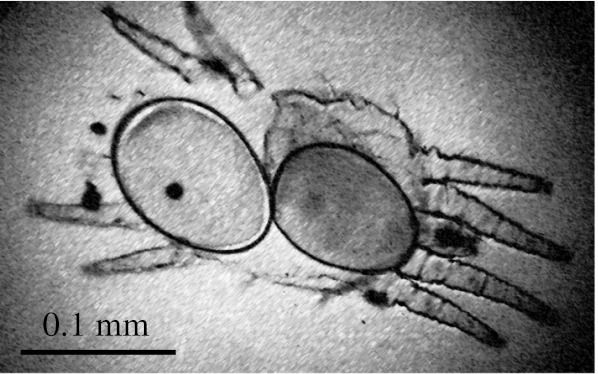
Paedogenesis occurring in a nymphal stage of *Brevipalpus phoenicis*. This was first reported by Baker (1972). Some *B. phoenicis* colonies maintained in the laboratory exhibited occasional events of paedogenesis

Paedogenesis in *Brevipalpus* mites was first reported by Baker (1972). Occasional events of sexual maturity of immatures were observed in some *B. phoenicis* colonies that were maintained in the laboratory (Fig. 2). There is no conclusive explanation for the occurrence of this phenomenon or its potential association with sex-alterating symbiont bacteria. Kennedy (1995) suggested that ‘phase variation’ (morphological, behavioral and physiological variations observed within species resulting in many cases of density effects during developmental stage), is another adaptive character associated with the *B. phoenicis* species complex; this phenomenon may explain the shortening of the developmental time of the mite under situations of high population density.

## Expansion of *Brevipalpus* transmitted viruses (BTVs)

*Brevipalpus* transmitted viruses (BTVs) are New World plant pathogens with one known exception, the *Orchid fleck virus* that is reported worldwide (Kondo et al. 2003). Citrus leprosis-like symptoms were reported to occur in South Africa, Philippines, China, India, Japan, Java and Sri Lanka (Fawcett and Lee 1926, Fawcett 1936), but the causal agent and its vector were not confirmed.

Over the past decade, the spread and economic importance of *Citrus leprosis virus* (CiLV) into new areas of South and Central America (including Belize) and most recently into southern Mexico has been alarming (Table 1). The spread of this disease across such a large area could be associated with the great increase in the number of hectares of citrus under production in the region (host density) as well as the movement of infected plants and mites from areas where the disease has established. The commonly known species of *Brevipalpus* mites that infest citrus in tropical and subtropical areas in the Western Hemisphere have a host range that includes hundreds of plant species. Concerns exist about the presence of host plants that are cryptic, asymptomatic, reservoir hosts that can sustain or magnify citrus leprosis-like viruses.

**Table 1:** The occurrence and spread of citrus leprosis through the Americas

Years	Country (places)	References
1880–1955	USA (Florida)	Fawcett (1936)
	USA (Mississipi)^a^	Bitancourt (1955)
1920	Argentina (Misiones, Corrientes)	Spegazzini (1920)
1920	Paraguay	Spegazzini (1920)
1933	Brazil (Sao Paulo)	Bitancourt et al. (1933)
1937	Brazil (Rio Grande do Sul)	Bitancourt and Grillo (1934)
1940	Brazil (Minas Gerais)	Bitancourt (1940)
1955	Brazil (Pará)	Bitancourt (1955)
1955	Brazil (Piauí)	Bitancourt (1955)
1993	Brazil (Rondonia)	Teixeira et al. (1993)
1999	Brazil (Tocantins)	Domingues and Rodrigues (1999)
1940	Uruguay	Bitancourt (1940)
1955	Bolivia	Bitancourt (1955)
1974	Venezuela	Rodrigues (1995)
1995	Guatemala	Mejia et al. (2002)
1995	Panama	Dominguez et al. (2001)
2000	Costa Rica	Araya Gonzales (2000)
2003	Colombia	Leon et al. (2006)
2004	Mexico (Chiapas)	SAGARPA (2009)
2005	Mexico (Tabasco)	SAGARPA (2009)
2006	Honduras	Rodrigues et al. (2007)
2008	Mexico (Oaxaca)	SAGARPA (2009)
2009	Mexico (Campeche)	SAGARPA (2009)
2011	Belize	Anonymous (2011)

Revised after Rodrigues et al. (1995) and Childers and Rodrigues (2011)

^a^No photos or further confirmation provided

**Fig. 3:**
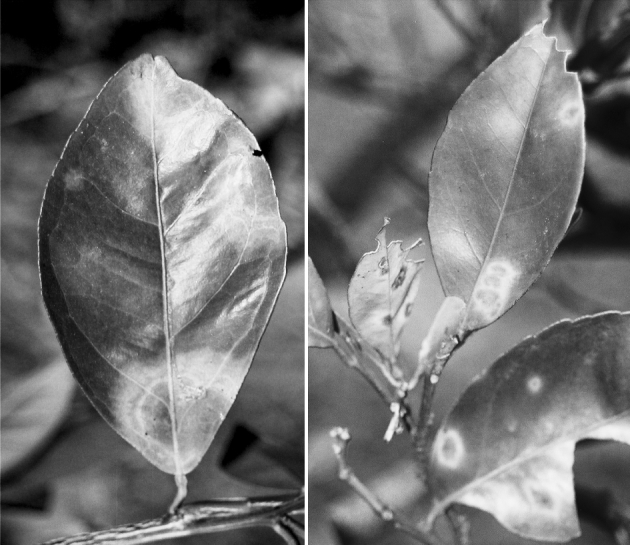
Symptoms of both the cytoplasmatic form of citrus leprosis (*left*, CiLV-C) and the nuclear form (*right*, CiLV-N) in sweet orange leaves. Symptoms of the N type of citrus leprosis were usually smaller than those of the C type. Also, the C type of citrus leprosis had an intense *yellow margin* around the chlorotic lesion

In Brazil, evidence that citrus leprosis was caused by a virus was presented by Kitajima et al. (1972). They identified the occurrence of nuclear (CiLV-N) virus particles associated with sweet orange leaf lesions that were collected from the field. The form of citrus leprosis that occurred in Florida (USA) prior to the 1960s was reported to be the nuclear type of citrus leprosis (Kitajima et al. 2011). Today, CiLV-N is rarely found in commercial citrus orchards in Brazil or elsewhere. The prevalent form of citrus leprosis that is spreading throughout Central America and Mexico is the cytoplasmic (CiLV-C) type (Fig. 3). Key questions remain to be answered: Which virus type was predominant in the past? What led to the near disappearance of the nuclear type and emergence of the cytoplasmic type of citrus leprosis? What factors could be associated with these changes? Where did citrus leprosis and related viruses originate? Are the two virus types from similar origins? Citrus, coffee and many other host plants that are widely grown in Central and South America are not originally from the Western Hemisphere. Are those viruses present within non-symptomatic host plants that serve as reservoirs of infection? Are the many BTVs reported by Kitajima et al. (2010) capable of being transmitted to other plants? Can the mite vectors switch host plants easily (Rodrigues et al. 2005, Nunes et al. 2012)? What role(s) do cryptic species of *Brevipalpus* that are co-habiting one or more of the hundreds of reported plant hosts play in virus transmission (Childers et al. 2003b, Childers and Rodrigues 2011). In Hawaii, Melzer et al. (2012) reported a leprosis-like disease infecting *Citrus volkameriana* Tan & Pasq and *Hibiscus* sp. It could be hypothesized that potential new variations of the viruses emerge because they are constantly evolving and interacting with new or more efficient *Brevipalpus* vector biotypes.

It has been assumed that the cytoplasmic form of citrus leprosis is more virulent than the nuclear form. This is a speculative assertion and is not supported by direct experimental evidence, but rather based primarily on the frequency of occurrence of characteristic foliar and fruit lesions of the two types in the field. Citrus leprosis reached Mexico around 2004 and spread rapidly through all the major southern citrus-growing areas. Mexico is known to have a rich fauna of false spider mites including many species of *Brevipalpus* (Baker and Tuttle 1987, Mesa et al. 2009). Recently, both the cytoplasmic and nuclear types of citrus leprosis were identified in Mexico (G. Otero-Colina, pers. comm., 2012). Is it possible that the introduction of both forms of citrus leprosis into southern Mexico overlapped with the new biotypes of mites vectors? This could result in a different pattern of disease spread. To better understand the movement of the disease, it is important to know whether the viruses and the vector biotypes are spreading together, or whether the virus is spreading by vector biotypes or species already occurring in the new region.

Complex pathogen-vector-host interactions such as citrus leprosis represent a challenge for states, countries and regions to effectively establish quarantine measures and barriers. This is despite growing efforts and collaboration among various research institutions and governmental agencies. Biological field studies of *Brevipalpus* mite populations are needed that include both taxonomic and molecular identification of mite populations not only on citrus but also on adjacent agricultural crops and associated ornamental and ground cover plants including weeds that may serve as host plants.

New mite vectors, virus-infected plants, or virus-infected *Brevipalpus* mites could arrive into the USA through various pathways (Childers and Rodrigues 2005). Therefore, a proactive approach would be more effective than relying on conventional interception or quarantine measures (Heather and Hallman 2008). A coordinated effort among US citrus producing states is needed to: (1) identify the *Brevipalpus* species occurring within and around citrus orchards in each state, and (2) to determine the potential of those *Brevipalpus* species to transmit one or both forms of CiLV. Determining the identity and biology of the *Brevipalpus* vector(s) and virus types that are spreading in citrus and alternate host plants through Mexico, Belize and other Central American countries is needed. Also, determining the timing, magnitude and dispersal distances of *Brevipalpus* vector species would provide invaluable information for more effective management capabilities than currently exist (Childers and Rodrigues 2011). Research is needed to minimize the further spread of this serious citrus disease and to reduce potential economic losses.

## Transmission of *Citrus leprosis virus* and vector fitness

**Fig. 4:**
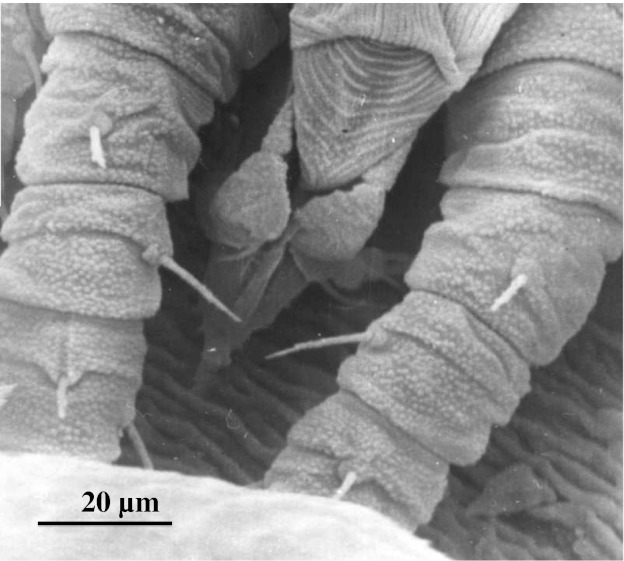
SEM image showing the moment an adult *Brevipalpus phoenicis* is piercing a citrus leaf. Both mite palpi are aligned to guide the stylets into the plant tissue

The *Brevipalpus* mite feeding process is crucial in virus acquisition and transmission success (Fig. 4). Two transmission assays were conducted using progeny obtained from a colony of mites established from a single female egg of *B. phoenicis* (Rodrigues et al. 2004). The original virus isolate used in this study was the cytoplasmic form of citrus leprosis as reported by Locali et al. (2003). Individual mites from the viruliferous colony were transferred to sweet orange seedlings (one mite/plant) and kept under controlled environmental conditions (25 °C, 12:12 light:dark) for 60 days, after which the seedlings were observed for the development of citrus leprosis symptoms. Individual mite survival and colony establishment exceeded 80 % with transmission rates of 10–11 %, for individual adult female mites (Rodrigues 1995). Previous studies conducted in Brazil by Chagas et al. (1983) and Chiavegato (1995) reported transmission rates of citrus leprosis by *Brevipalpus* mites to be about 8 %. However, neither the transmitted virus type of citrus leprosis nor the *Brevipalpus* vector species were identified in those studies.

A clonal mite population of *B. phoenicis* (GenBank Accession AY320019) was divided and maintained over an 18 month interval to assess feeding on both citrus leprosis-infected and healthy tissues. This was done to verify the influence of the cytoplasmic type of citrus leprosis on *Brevipalpus* fitness. Fitness—evaluated by egg quality through the frequencies of larval hatching—was not affected by the occurrence of citrus leprosis in the mite colony (Table 2).

**Table 2:** Accumulated frequencies observed (Fo) and expected (Fe) of *Brevipalpus phoenicis* larvae hatching from colonies associated with citrus leprosis (CoAL, n = 90 eggs) and without citrus leprosis (CoNAL, n = 223 eggs)

Days	CoAL		CoNAL		Chi-square (ns)
	Fo	Fe	Fo	Fe	
1	2	2.9	8	7.1	0.15
4	10	11.8	31	29.2	0.44
7	19	22.1	58	54.9	0.83

Symbionts could influence vector behavior and directly or indirectly affect the ability to acquire or transmit the pathogen during the feeding process. *Cardinium* bacteria were reported to infect *Brevipalpus* populations (Weeks et al. 2001) but were shown not to affect the fitness of *B. californicus* mites associated with the transmission of orchid fleck virus in orchids (Chigira and Miura 2005).

Vector-transmitted parasites react to and induce changes in their environment both in the host and vector, as well as in response to other parasites (Matthews 2011). However, very little is known about the molecular and physiological changes in plant hosts and vectors during the process of infection by BTV’s. Considering the physiological changes in leprosis-infected tissues, Nogueira et al. (1996) reported higher levels of iron among other elements associated with citrus leprosis lesions. This was explained by the higher accumulation of ferritin-like arrays associated with pro-plastids on citrus infected cells (Rodrigues 1995). Freitas-Astua et al. (2007) reported the influence of viruses on the expression of plant genes related to plant energy and metabolism in the earlier stages of infection. This agrees with previous cytopathological observations (Rodrigues 1995). In addition, differential host susceptibility to citrus leprosis among citrus species, hybrids and varieties, as shown with ‘Sabará’ tangor and grapefruit could play important roles in virus persistence (Rodrigues 2006).

## Social, environmental and economic impacts of *Brevipalpus* transmitted diseases

**Fig. 5:**
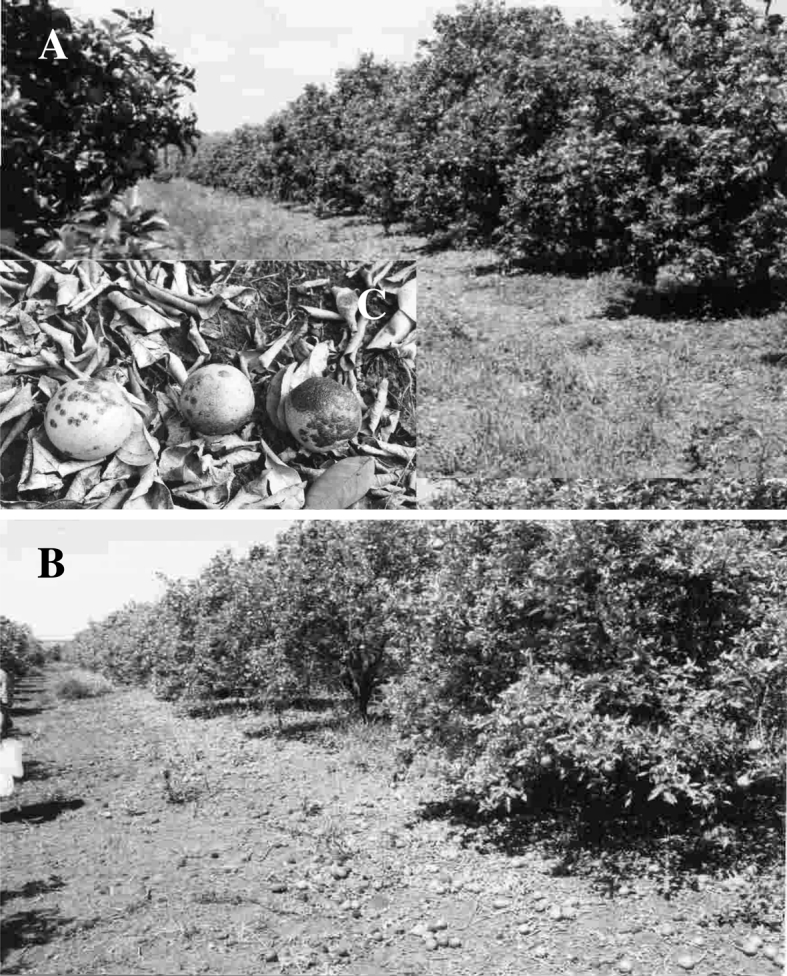
**a** Sweet orange orchard showing initial symptoms of leprosis. **b** The following year the same trees had severe dieback, premature leaf and fruit drop caused by citrus leprosis. **c** Symptomatic damage by the disease to fruits and leaves that occurred in the Artur Nogueira municipality, SP, Brazil

The first author visited El Salvador, Central America, in 2010 and met various growers in a citrus cooperative. The group was asked about their major problems in growing citrus and the common answer was ‘mites’. This appeared to be due to the recent introduction and subsequent damage caused by citrus leprosis. A similar situation happened in previous years in Brazil where excessive citrus fruit losses resulted from inability to manage citrus leprosis (Fig. 5; Table 3). Sweet oranges are an important food staple and a major source of vitamins for rural communities throughout Central and South America. Citrus also provides a ready-cash crop for small growers in these countries that sell their produce in local urban markets.

**Table 3:** Mean yield (weight and number of fruits per tree) on 3-year-old sweet orange varieties (six plants/variety) and Palestine sweet lime infested with citrus leprosis-infected *Brevipalpus* mites, in 1999, Piracicaba, SP, Brazil

Variety	Weight (kg/ha)		Nu. fruits/tree		% fruits with symptoms	
	Infested^a^	Control^b^	Infested^a^	Control^b^	Infested^a^	Control^b^
Palestine lime	13,057	9,408	510	360	0	0
Valencia	5,572	6,206	178	190	64	21
Pera Rio	4,643	7,373	123	217	85	31
Lima	2,580	3,600	67	108	57	6
Bahia	648	2,226	19	69	73	19
Seleta	8	5,245	1	132	100	14
Barão	812	1,264	34	49	71	14
Natal	4,335	10,668	169	360	98	47
Hamlin	2,076	9,356	65	313	83	7

^a^After 18 months in the field, each plant was infested with 50 adult females that originated from a single female colony, which was kept on infected CiLV-C sweet orange seedlings

^b^Plants were not infested with mites and received monthly acaricidal sprays for 8 months

**Fig. 6:**
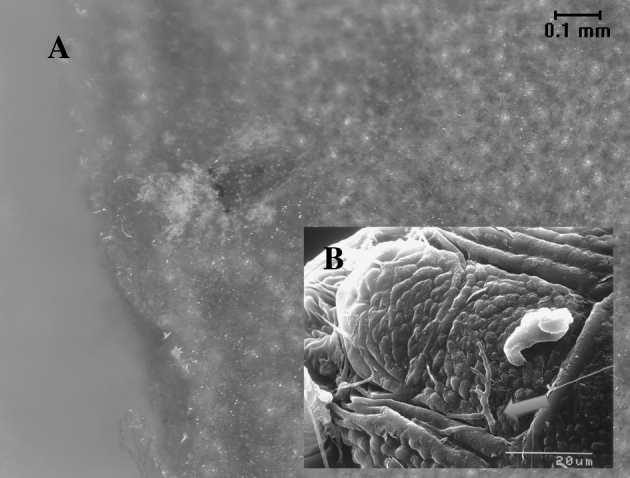
Microbial pathogens are major natural enemies suppressing *Brevipalpus* populations. *Metarhizium* is one of the fungi reported to infect *B. phoenicis* as shown in (**a**) (Magalhães et al. 2005). SEM (**b**) shows fungal hyphae (*arrow*) growing between the opisthosomal plates

Production costs to control *Brevipalpus* mites in sweet orange orchards in the state of São Paulo ranged from 12 to 30 % of total production costs or 29–89 % of total phytosanitary expenses (Rodrigues et al. 2002). This does not include losses and costs of eventual pruning when citrus leprosis occurs on tree limbs or branches. Current control approaches for citrus leprosis include progressive inoculum reduction strategies in citrus orchards through scouting and acaricidal spray applications for the mite vector. Using such an integrated approach, losses caused by the disease can be reduced and efficiency of vector control can be enhanced with natural enemies (Fig. 6). Table 3 summarizes yields from various sweet orange varieties (Pera Rio, Valencia, Natal, Hamlin, Seleta, Barão, Lima and Bahia) and Palestine Lime (*Citrus limettiodes* Tan., asymptomatic to CiLV) that either received or did not receive acaricide sprays. The results were highly variable and were influenced by the orange variety, cost of the control package adopted and potential return to the grower (Dragone et al. 2003). Palestine sweet lime was shown to be immune to CiLV-C and was the only variety that was not negatively affected by the vector-citrus leprosis combination. All sweet orange varieties were severely affected by citrus leprosis in different degrees. In addition to citrus leprosis lowering yields (kg/ha), the remaining fruits on the tree had leprosis symptoms ranging from 57 to 100 % on infested trees compared with 6–47 % on plants that received acaricide sprays. Considering percentage of fruits showing leprosis symptoms, sweet orange varieties Natal, Pera and Seleta were more susceptible, followed by Hamlin, Baía, Valencia, Barão, Lima and Lima Verde. The varieties also showed differential responses to the acaricide treatments.

Other hosts plants with BTV’s including coffee, passion fruit and many ornamental plants have been reported by Kitajima et al. (2010) that included new areas of citrus introductions into both agricultural and natural environments (Rodrigues et al. 2008) and urban settings (Miranda et al. 2007). In many instances the initial spread of citrus leprosis goes unnoticed while both movement of people and goods occur.

## Conclusions

*Brevipalpus* mites and viruses that they transmit are a growing phytosanitary threat to the citrus industries of the world. The mites themselves are intriguing organisms in terms of their genetic uniqueness (haploid females showing two non-homologous chromosomes), occurrence of symbionts affecting their sex ratios and the close relationship with numerous cytoplasmic and nuclear types of viruses. To date, these mite-vectored viruses include only non-systemic pathogens that persist under natural conditions and represent an increasing major economic, environmental and social threat to agricultural and ornamental industries.
